# Foodservice interventions and their influence on nutritional outcomes and satisfaction of adult oncology patients—a conceptual replication

**DOI:** 10.1007/s00520-025-09264-5

**Published:** 2025-02-24

**Authors:** Belinda Steer, Jacqueline Black, Laura Cecchini, Clarissa Choo, Ana Dvarakesa, Martha Hill, Athena Ho, Georgia Kennett, Andre Woltersdorf, Emily Zilber, Judi Porter

**Affiliations:** 1 Nutrition and Speech Pathology Department, Peter MacCallum Cancer Centre, Melbourne, Australia; 2 School of Exercise and Nutrition Sciences, Deakin University, Geelong, Australia; 3 School of Exercise and Nutrition Sciences and Institute for Physical Activity and Nutrition, Deakin University, Geelong, Australia

**Keywords:** Foodservice, Adult, Oncology, Conceptual replication

## Abstract

**Background:**

Foodservices are vital components of care for oncology patients across all settings. This conceptual replication aimed to explore the influence of foodservice interventions across a range of nutritional outcomes and the satisfaction of hospitalised and ambulatory adult oncology patients.

**Methods:**

The review protocol was registered with PROSPERO (CRD42023447351). Six databases were searched using search terms associated with the intervention and population. There were no restrictions on language or publication date. The inclusion criteria were applied to titles and abstracts, then full-text papers. All included papers were assessed for study quality. Outcome data were synthesised narratively, and vote counting was conducted based on the direction of effect.

**Results:**

Eight studies testing the influence of a foodservice intervention were included. Interventions included technology advancement, high energy high protein meals and snacks, and patient-focused foodservice models. Study quality was assessed as positive for seven of the studies. Of nutritional outcomes reported (energy and protein intake, body weight, muscle mass), improvements were seen in all but one study. Except in one study, where reported, patient satisfaction improved as a result of the intervention.

**Conclusion:**

Original research into the impact of systematic foodservice interventions has expanded in recent years. This conceptual replication found that the implementation of small, frequent high-energy high-protein meals and snacks, and patient-focused foodservice models may improve nutritional outcomes and satisfaction in both inpatient and ambulatory settings. Further large-scale research that explores multiple interventions and cost-effectiveness is required.

**Supplementary Information:**

The online version contains supplementary material available at 10.1007/s00520-025-09264-5.

## Introduction

The menu and foodservice are integral components of the care that patients receive during a hospital admission. Menu interventions are also a key aspect of nutrition care delivered to ambulatory patients. In addition to nutritional considerations, there are multiple other reasons to ensure that the foodservice system meets the needs of the patients, including its role as a significant determinant of satisfaction [1] and its contribution to waste [2, 3]. Foodservice is a crucial component to improving patient health outcomes particularly in oncology settings since many patients are nutritionally compromised at the time of admission, whilst some develop malnutrition during their inpatient stay [4]. Foodservice operations are typically described as utilising a systems approach, whereby inputs are transformed into outputs with interrelationships throughout the system [5].

Oncology patients are at an increased risk of malnutrition [6]. This is complicated by challenges that may impact the patients’ ability to orally ingest adequate intake of food due to limiting factors such as functional and cognitive impairments and/or physical site of tumour, particularly among those with head and neck cancers [7]. Further, some patients experience nutritional decline due to side effects of treatment such as loss of appetite, dysgeusia, dysphagia, xerostomia, mucositis, odynophagia, trismus, and nausea [8, 9]. Changes in taste and satiety are commonly experienced by cancer patients undergoing treatment with chemotherapy, radiotherapy, or surgical procedures [7]. The utilisation of appropriate nutrition and hospital foodservice interventions is a key course of action towards reducing nutritional decline and malnutrition prevalence [10]. Delivery of these interventions in an effective manner can have positive impacts on patient treatment and recovery therefore improving patients’ quality of life through patient satisfaction and experience.

The hospital foodservice system is known to be heavily reliant on oral nutrition support (ONS) in addition to hospital meals to meet the requirements of patients [11, 12], highlighting the importance of appropriate hospital foodservice models designed to provide meals of high-quality nutrients effectively [13]. Foodservice models adopted within hospitals and healthcare services are not uniform, leading to a variable level of care provided to patients.

A previous review [14] examined the influence of foodservice interventions (including ONS interventions) in oncology settings on a range of clinical outcomes. However, due to a limited range of foodservice interventions, the previous [14] review focused primarily on research covering the impacts of ONS across a range of outcomes. In recent years, there has been an expansion in the research base investigating interventions in foodservices for oncology patients. This has enabled the research team to undertake a conceptual replication of the original review. Such a replication is warranted for several reasons, including “lack of clarity about the technical or statistical methods or the judgments made, such as the subjective decisions related to defining criteria for inclusion of the population, intervention or exposure, and outcomes of interest; and data collection, synthesis, and interpretation” [15]. Therefore, this conceptual replication aimed to explore the influence of foodservice interventions across a range of nutritional outcomes and the satisfaction of hospitalised and ambulatory adult oncology patients.

## Methods

The Preferred Reporting Items for Systematic Reviews and Meta-Analyses (PRISMA) guided the reporting of the review [16]. This conceptual replication was registered with the International Prospective Register of Systematic Reviews on 31 July 2023 (CRD42023447351).

### Eligibility criteria

In this update, the inclusion criteria were revised due to the expansion of the evidence base with a focus on foodservice interventional studies alone. Consequently, trials investigating interventions focused on ONS products and education, dietary counselling, and parenteral and enteral nutrition, were ineligible.

The population of interest was hospitalised and/or ambulatory adult oncology patients aged ≥ 18 years. Eligible studies prospectively tested foodservice interventions such as menu and service modification or enhanced eating environments with limited or no ONS assistance or nutrition counselling. The comparator group was not defined a priori, but could utilize a prospective control arm, participants as their own controls (pre-post studies), or retrospective controls with a focus on usual or standard care. Primary outcomes consisted of nutritional (energy and protein) intake, nutritional status, and body weight change. Plate waste and patient satisfaction were secondary outcomes extracted from included studies. Full-text studies of original research of any study design except retrospective audits were considered. Conference abstracts, narratives, commentaries, and reviews were not eligible.

### Information sources and search strategy

The search strategy and approach to study identification were replicated from the review of Doyle et al. [14] to identify recent studies investigating the influence of foodservice interventions on adult oncology patients.

Six electronic databases were searched as follows: Ovid MEDLINE (R) Epub Ahead of Print, In-Process and Other Non-Indexed Citations, Ovid MEDLINE (R) Daily and Ovid MEDLINE (R) 1946 to present; PsycINFO (Ovid); Informit Health Collection & Informit Humanities and Social Sciences Collection; CINAHL Plus with full text (EBSCO Host); EMBASE (Embase.com) and the Cochrane Library. There were no restrictions on language; searches dates were July 2016 (to update from the previous search that had no search restrictions) to 5 March 2023. Details of the search conducted in Ovid Medline are shown in Supplementary file 1. This search strategy was adapted as necessary for the other five databases. Database searches were exported to Covidence (Veritas Health Innovation, Melbourne).

### Selection process

After the removal of duplicates both by algorithm and manual identification in Covidence, title and abstract screening was undertaken independently and in duplicate by the research team. No machine learning was used in the screening process. Two reviewers also screened full-text papers, with conflicts assessed by an independent third author.

### Data collection process and data items

A customised data extraction tool was developed by researchers and included the following key data: type of study performed, details of the intervention(s) and comparator (where applicable), duration of the intervention, the sample population, and eligible outcomes of the intervention. Additional study characteristics extracted were as follows: author/date/country of research and sample size/retention. Where required, outcome data were extracted from graphs using visual estimates. Data extraction was undertaken independently and in duplicate; no automation tools were used. Only published data were extracted; data were not obtained or confirmed from study investigators. Where there were multiple timepoints, the intervention final available timepoint was selected. *p* values were extracted as per the original study.

### Study risk of bias assessment

Study quality was assessed by two reviewers (including one reviewer assessing all records) using the Quality Criteria Checklist [17]. This quality assessment tool is specific for studies in nutrition and dietetics and facilitates the evaluation of study relevance and validity across multiple study designs. Validity question (where applicable) considers the following aspects: the research question, bias in the selection of participants, comparability of groups, handling of withdrawals, blinding, detail of reporting of exposures/procedures, validity/reliability of measurements, appropriateness of statistical analysis, justification for study conclusions, and influence of funding or sponsorship. Included studies were evaluated across each component of the tool, with a final rating determined according to the tool guidelines. The Cochrane risk of bias tool used in the previous review [14] was not used due to the heterogeneity of study designs.

### Synthesis methods

The narrative synthesis includes a brief description of the studies identified (including study design, size, and population demographics). Narrative synthesis occurred by sub-group per outcome. There was considerable heterogeneity in the intervention and comparator arms and in the outcomes reported. Consequently, a meta-analysis of findings could not be undertaken. Instead, the effects were described using vote counting based on the direction of the effect. The approach taken to vote counting was guided by the Cochrane Handbook [18]. An effect estimate from the intervention arm(s) was categorised as showing benefit (e.g., a positive effect = increased energy intake) or harm (e.g., a negative effect = decreased energy intake), based on the observed direction of effect, irrespective of whether the difference was statistically significant at *p* < 0.05. Harvest plots were developed to represent the results of the primary outcomes.

## Results

### Study selection

**Fig. 1:**
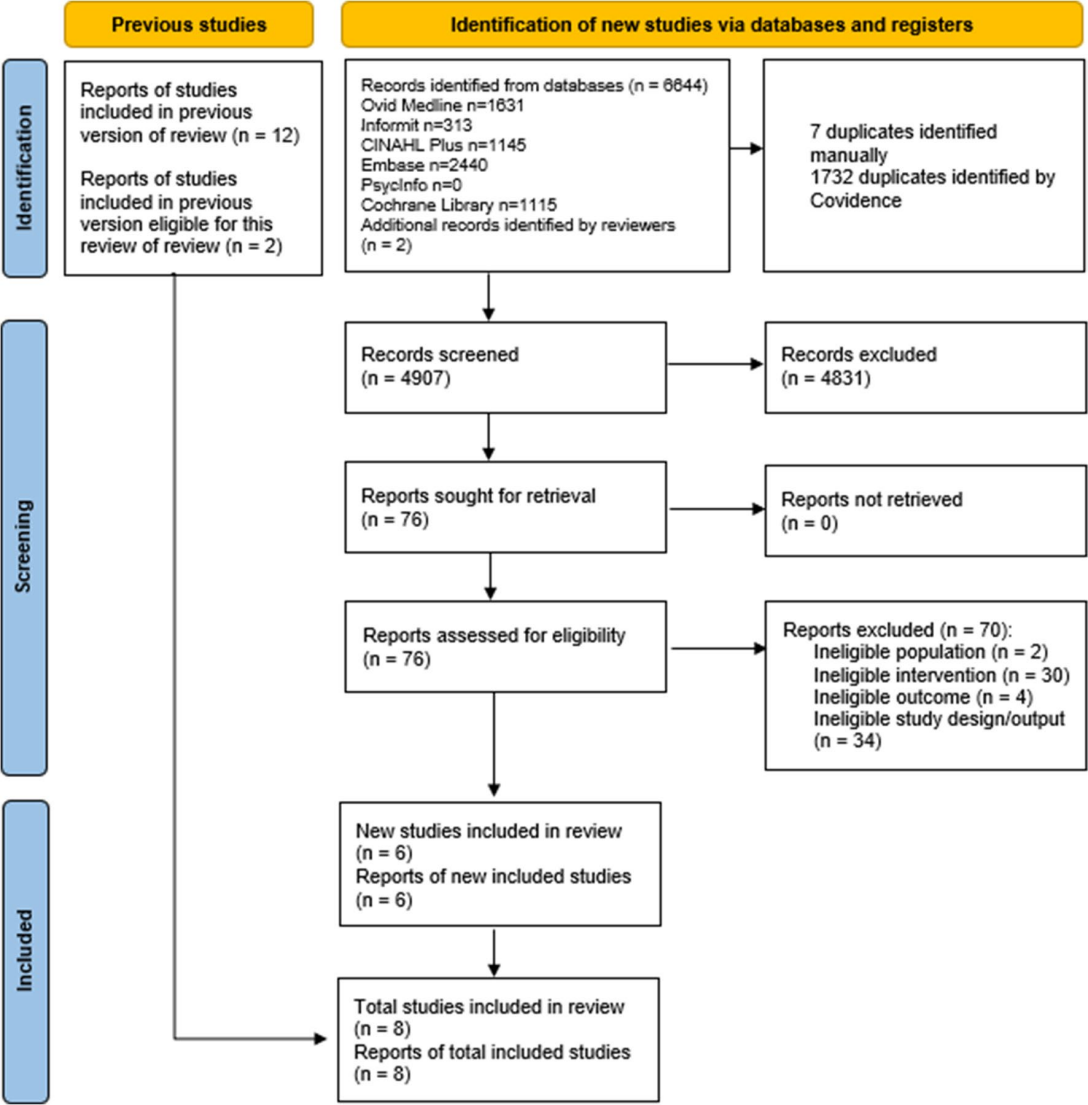
Flow diagram of study identification

The total yield from all databases was 6644 results, reduced to 4907 after the removal of duplicates. Following the full-text review, six studies were identified that met the eligibility criteria (Fig. 1); these were added to the two studies identified in the previous review [14]. Studies excluded during the screening of full texts are listed in Supplementary file 2.

### Study characteristics

The eight included studies are shown in Table 1. A variety of study designs including observational studies [19], non-randomised studies of interventions [20–24], and randomised controlled trials [25, 26] met the eligibility criteria. Participant numbers were relatively small, ranging from 27 [23] to 201 [19] participants. Study duration was also typically brief and ranged from 2 weeks [19] to 4 months [24]. There were no studies that evaluated a foodservice intervention at scale and over an extended period in this setting. The majority of studies were focused in the inpatient setting [19, 21, 23, 24]; however, several sought to evaluate menu interventions in ambulatory settings [25, 26] or multiple settings [20, 22].

**Table 1:** Characteristics of studies investigating foodservice interventions in adult oncology patients

Author, date, country	Study design	Population	Sample size; retention	Brief description of the intervention	Brief description of the comparator	Intervention duration	Outcomes of interest
Barrington et al., 2018, Australia	Observational study	Hospitalised oncology patients	*n* = 201 (*n* = 105 intervention; *n* = 96 comparator) Meal experience survey: *n* = 50 participants intervention; *n* = 58 comparator All inpatients admitted during the study period were eligible; study retention was not reported	24-h bedside menu ordering system	Traditional paper-based menu	2 weeks	Energy intake, protein intake, patient meal experience, plate waste
Bille et al., 2018, Denmark	Non-randomised study of intervention	Hospitalised and ambulatory haematological cancer patients; Participants hospitalised for a mean of 4 days (out of 4/52) in each period	*N* = 121 invited; *n* = 32 participants completed; study retention not reported	A choice of 4 high-energy, high-protein meals during admission Participants prepared food at home or purchased similar meals in supermarkets	Habitual diet	8 weeks	Weight change
Ijmker-Hemink et al., 2023, Netherlands	Randomised controlled trial	Ambulatory cancer patients receiving chemotherapy	*n* = 148 (*n* = 72 intervention, *n* = 76 control) *n* = 96 retained (*n* = 50 intervention, *n* = 46 control)	5–6 small protein-rich meals delivered to the home, excluding breakfast and drinks	Usual diet	3 weeks	Quality of life, energy and protein intake, patient satisfaction
Leedo et al., 2017, Denmark	Randomised controlled trial	Ambulatory patients with lung cancer as a primary cancer	*n* = 40 (*n* = 21 intervention, *n* = 19 control) *n* = 38 retained (95%) (*n* = 20 intervention, *n* = 18 control)	High-energy, high-protein main meals and snacks via hospital meals and snacks delivered to the home. Patients could order one main meal/day and snacks at the libitum. Ordered via a tablet with an illustrated online menu	Usual diet	12 weeks	Energy and protein intake, body weight change
Lindman et al., 2013, Denmark	Non-randomised study of intervention	Hospital inpatients > 18 years diagnosed with haematological cancer receiving complex curative chemotherapy or aiming at long-term survival	*N* = 99 participants in food intake study; *n* = 152 participants completed the questionnaire; 87% retained	Kitchen assistants trained as food caregivers. Additional tasks (in addition to usual tasks): address the patients and their relatives directly once a day; serve snacks; guide patients and relatives; encourage and motivate patients to eat	Kitchen assistants working in the kitchen on the wards performing usual tasks	Patient food intake was recorded for 3 days. Evaluation was conducted 3 times before and after the intervention	Energy and protein intake, patient satisfaction
Nguyen et al., 2021, Vietnam	Non-randomised study of intervention	Hospitalised and ambulatory patients 18 years diagnosed with stomach or colon cancer receiving chemotherapy	*N* = 120 (*n* = 60 intervention, *n* = 60 control) *n* = 103 retained (*n* = 50 control, *n* = 53 intervention)	Nutrition counselling and a specific menu designed to meet individual energy and protein requirements	Menu based on individual demands	2 months	Nutritional status
Pietersma et al., 2003, Canada	Non-randomised study of intervention	Hospital inpatients admitted to an acute oncology/palliative care unit	*N* = 27 participants (including *n* = 23 people with cancer) 82% of participants completed	Lunch is delivered on an electric food cart. Participants received the same food as provided by tray service but the food cart enabled patients to choose meal at point of service	Breakfast and supper are provided by tray service	10 days	Patient satisfaction
Sathiaraj et al., 2019, India	Non-randomised study of intervention	Hospitalised oncology patients	*n* = 160 (*n* = 100 patient centred foodservice, *n* = 60 in traditional foodservice) Attrition not reported	Patient-centred foodservice model	Traditional foodservice model	4 months prospective versus 3 months retrospective component	Patient satisfaction, energy, and protein intake

### Results of individual studies

There were multiple different foodservice interventions tested in the oncology population. These included innovations in technology [19, 26], choice of high-energy and protein main meals or snacks [19, 22, 25, 26], and a greater patient focus within the foodservice model including changes to the food delivery/distribution or level of staff support [21, 23, 24].

The effect of interventions varied between studies, particularly those where the interventions were heterogeneous. Statistically significant increases both in energy (*p* < 0.001) and protein (*p* < 0.001) intake were reported in the 2-week 24-h bedside menu ordering system study of Barrington et al. [19] and the 4-month patient-centred foodservice model study of Sathiaraj et al. [24]. In comparison, no statistically significant findings were reported for these outcomes in the 12-week trial of home-delivered high-energy, high-protein main meal and snacks study of Leedo et al. [26]. Further study-specific details are reported in Tables 1 and 2.

**Table 2:** Eligible outcomes from studies investigating foodservice interventions in adult oncology patients

Outcome	Author, year	Intervention results	Control results	*p* value
Energy intake	Barrington, 2018	Mean 6457, SD 3069 kJ/day	Mean 4805, SD 2028	< 0.001
	IJmker-Hemink, 2023	Relative to requirements energy intake was 21% higher in the intervention group. Energy requirements met in 67% of participants	Energy requirements met in 35% of participants	< 0.05
	Leedo, 2017	Mean 1758, 95% CI (− 377 to 3982) kJ/day intervention–control		0.35
	Lindman, 2013	Met an average of 93.3% of estimated energy requirements (CI 95% 82.3–104.3)	Met an average of 76.2% of estimated energy requirements (CI 95% 64.6 to 87.9)	0.03
	Sathiaraj, 2019	Mean 6827, SD 660.9 kJ/day	Mean 6276, SD 715.7 kJ/day	< 0.001
Protein intake	Barrington, 2018	Mean 72.3, SD 36.7 g/day	Mean 57.7, SD 26.9 g/day	< 0.001
	IJmker-Hemink, 2023	Intake relative to requirements is similar in both groups		NS
	Leedo, 2017	Mean 15.8 (95% CI − 5.6 to 37.1) g/day (intervention–control)		0.38
	Lindman, 2013	Met an average of 69.1% of estimated protein requirements (CI 95% 59.6–78.5)	Met an average of 64.3% of estimated protein requirements (CI 95% 53.7 to 75.0)	0.51
	Sathiaraj, 2019	Mean 59.89, SD 10.9 g/day	Mean 48.42, SD 10.8 g/day	< 0.001
Change in body weight	Bille, 2018	Mean 1.2, SD 1.9	Mean –2.8, SD 5.2	0.0008
	Leedo, 2017	Mean 0.8, 95% CI (− 1.7 to 3.4) kg intervention–control		0.69
Weight and muscle mass	Nguyen, 2021	Weight and muscle mass had statistically significant improvements in the intervention but not control groups		NR
Nutritional status (PG-SGA) PG-SGA A PG-SGA B PG-SGA C	Nguyen, 2021	*N* (*n* = 53) 34 14 5	*N* (*n* = 50) 20 21 9	NR
Patient satisfaction	Barrington, 2018 Understanding of menu Completion Received food ordered Selected liked foods Food choice is difficult Meal timing	% 66 87 74 32 20 Food is as ordered 87	% 65 55 48 61 33 55	0.96 0.0002 0.006 0.002 0.14 0.0005
	IJmker-Hemink, 2023	No differences between the groups Data not reported		NS
	Pietersma, 2003	Intervention arm received higher mean scores for all aspects of the patient satisfaction survey		Results of 6 of 7 questions were significantly higher for the intervention arm compared with the control (statistical analysis NR)
	Sathiaraj, 2019 Food quality Timeliness of delivery Flavour Therapeutic diet explanations Overall satisfaction	% 35.2 37.1 37.1 41.9 42.9	% 28.6 36.2 21.9 41 36.2	Results of all 5 patient satisfaction domains were higher for the intervention arm compared with the control (statistical analysis NR)
Plate waste	Barrington, 2018	Mean 34.3, SD 4.9% daily total	Mean 35.3, SD 4.5% daily total	0.75

*NR* not reported, *NS* not significant

### Results of syntheses

**Fig. 2:**
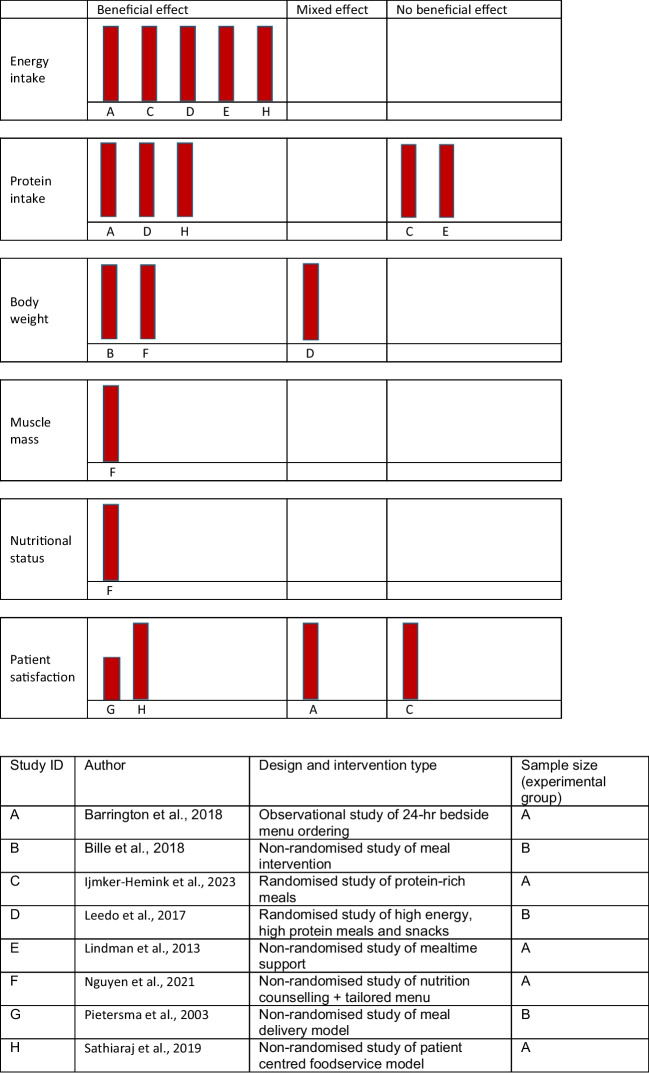
Harvest plot of primary outcomes. Sample size (experimental group): A, *n* > 50; B, *n* = 20–50; Study quality presented by bar height where taller bars represent higher quality studies

Key outcomes results are shown in Table 2 and presented as a Harvest plot in Fig. 2. Energy intake was improved in all five studies reporting on this outcome [19, 21, 24–26]. With the exception of the study by IJmker-Hemink [25], all studies reporting protein intake also showed improved intakes [19, 21, 24, 26]. Where reported, body weight improvements were greater in the intervention than in the control arms [20, 26], and muscle mass also improved [22].

Of the secondary outcomes, patient satisfaction improved in three [19, 23, 24] of the four foodservice interventions [25] where this outcome was reported. Measures to assess patient satisfaction were variable, and included the Kings College Food Service Questionnaire (27 items considering hospital patient foodservice experience) [19]; questionnaires considering well-being, shopping, cooking stress levels, and satisfaction with aspects of the meal service [25]; an investigator-developed questionnaire (11 items focusing on aspects of patient satisfaction and preferences) [23]; and an organisational tool for assessing patient satisfaction survey (5-foodservice domains) [24]. Only one study reported on wastage associated with the intervention foodservice model [19]; this was slightly lower in the intervention arm.

### Reporting biases

Except for one study [23], the quality of the selected methods and associated reporting was assessed as being positive (Table 3). Challenges in blinding within nutrition studies were consistently acknowledged. Despite the practical challenge of blinding patients and carers to a nutrition intervention, key outcomes were not assessed by independent members of the research team across the included studies. Study quality is also presented in Fig. 2 by bar height, where taller bars represent higher-quality studies.

**Table 3:** Quality assessment of included studies^a^

Author	Validity rating^b^										Overall rating
	N1	2	3	4	5	6	7	8	9	10	
Barrington, 2018	Y	Y	Y	N/A	N	Y	Y	Y	Y	Y	Positive
Bille, 2018	Y	Y	Y	N/A	N	N	Y	Y	Y	Y	Positive
IJmker-Hemink, 2023	Y	Y	Y	Y	N	Y	Y	Y	Y	Y	Positive
Leedo, 2017	Y	Y	Y	Y	N	Y	Y	Y	Y	Y	Positive
Lindman 2013	Y	Y	Y	Y	N	Y	Y	Y	Y	Y	Positive
Nguyen, 2021	Y	Y	Y	Y	N	Y	Y	Y	Y	Y	Positive
Pietersma 2003	Y	Y	Y	Y	N	N	N	U	Y	Y	Neutral
Sathiaraj, 2019	Y	Y	Y	N/A	N	Y	Y	Y	Y	Y	Positive

*Y* response of “yes” to the validity question, *N* response of “no” to the validity question, *U* unclear, *N/A* not applicable

^a^Assessed using The Quality Criteria Checklist for Primary Research

Relevance questions were positive for all studies. Validity questions: [1] research question stated; [2] participant selection free from bias; [3] comparable study groups; [4] method for withdrawals described; blinding used; [5] interventions described; [6] outcomes stated, measurements valid and reliable; [7] appropriate statistical analysis; [8] appropriate conclusions, limitations described; [9] funding and sponsorship free from bias. In addition, validity items 1–4 must be satisfied for a positive quality rating

## Discussion

This conceptual replication sought to examine the impact of foodservice interventions on a variety of nutritional outcomes and patient satisfaction in both hospitalised and ambulatory oncology settings. The replication of the original review was justified as the research question remains a priority for clinical practice indicated by an expansion in the evidence base since the original review. Nutritional decline and malnutrition in oncology settings remain a “wicked problem” [27], and broad foodservice systems approaches provide an opportunity to improve nutritional intake that complements individual nutrition care. This conceptual replication delivers more accurately on the interventions as proposed in the original review. Findings suggest that the implementation of foodservice interventions is likely to have a positive impact on nutritional outcomes. In particular, implementing smaller, more frequent high-energy, high-protein meals [20, 22, 25, 26] or more patient-focused foodservice models [19, 21, 24] may be promising approaches to improve nutritional outcomes in oncology patients.

Whilst the concept of small, frequent high-energy, high-protein meals is not new in the field of oncology foodservice, it is worth noting that the previous systematic review, conducted by Doyle et al. in 2017 [14], found no studies investigating this intervention, indicating that it represents a relatively recent advancement in the research literature. Interventions identified in the previous review relied predominantly on the addition of oral nutrition support products to increase nutritional intake and improve nutritional outcomes. This approach is reliant on individuals receiving and consuming an appropriate oral nutrition support product, compared to the interventions identified in this conceptual replication that focused on systematic interventions that could benefit all without the need for additional intake. Clear beneficial effects were identified in the included studies.

Although not previously published in oncology populations, the fortification of patient meals to increase energy and protein intake, and/or the addition of between-meal snacks, has been found to improve nutritional intake in other patient populations. A systematic review conducted by Mills et al. [28] examinedlooked at the evidence for using food fortification and snacks in older hospitalised patients. Eight articles compared at food fortification interventions  with usual nutrition care and found these interventions to be well tolerated, effective at improving dietary intake, and cost-effective. An earlier systematic review explored the influence of ONS interventions in rehabilitation settings and found similar results with the addition of higher energy meals, or smaller, fortified meals to the menu having an overall positive effect on nutritional intake [29]. The studies included in this conceptual replication demonstrate that there is a growing body of evidence reporting that these interventions are also effective in the oncology setting and can result in positive nutritional and patient outcomes.

As an alternative to specialised diets, different foodservice models have been investigated as a method to improve oncology patients’ nutritional intake, status, and satisfaction. Implementing foodservice interventions that make choosing, preparing, and/or consuming meals easier for all patients was shown to result in enhanced clinical outcomes. Studies included in this conceptual replication found that implementation of more patient-focused foodservice models, including 24-h access to bedside meal ordering [19], contribution of food caregivers [21], food cart meal delivery [23], a la carte menus [24], and home meal delivery [25, 26] had a positive influence on nutritional intake and outcomes for both hospitalised and ambulatory patients.

Heterogeneity in the methods used to evaluate the satisfaction of participants with different interventions makes comparisons from this perspective difficult. Despite this, results from a patient perspective were positive in the majority of studies where this outcome was measured. The use of validated foodservice satisfaction tools in the future would enable comparison between interventions and populations.

Notably, a study not eligible for this conceptual replication examined the influence of food delivery services for inpatients finding that the intervention group exhibited higher mean daily energy intake compared to the control group [30]. Patients in the intervention group reported higher satisfaction with the “Meals on Wheels” delivery service which was attributed to the freedom of ordering food when hungry and the ability to select foods based on their cravings, thus enhancing overall satisfaction [30]. This study was ineligible due to not having an oncology-specific focus, but the findings suggest that novel foodservice approaches introduced in other settings or populations may bring benefit to oncology inpatients in the future.

The strengths of this review were that there were no language restrictions, and a broad search strategy was applied across six databases to ensure all eligible studies were included. Due to the heterogeneity of the papers included, a meta-analysis was not possible, limiting the conclusions that can be drawn; however, the overall positive rating found in seven of the eight quality assessments suggests the appropriate and replicable methods were utilised. However, the authors acknowledge that the risk of bias assessment tool may overestimate the quality of the evidence, which is a consideration in results interpretation. Additionally, this conceptual replication focused on interventions in oncology populations only, potentially limiting the application of the findings to other populations.

In conclusion, this conceptual replication found that research into foodservice interventions in oncology settings has progressed beyond the provision of ONS to complement usual foodservices, to now be investigating the impact of systemic foodservice interventions that can benefit all oncology patients. The implementation of small, frequent high-energy high-protein meals and snacks and patient-focused foodservice models was found to improve a number of nutritional outcomes in both inpatient and ambulatory settings; however, further research is required to confirm these findings.

Larger studies are required, and future foodservice research should consider combining interventions to establish if additional benefits can be achieved. Given the focus on cost savings in healthcare, future studies should include a cost-effectiveness evaluation to determine if cost savings can be made when appropriate foodservice interventions are in place.

## Supplementary Information

Below is the link to the electronic supplementary material.

Supplementary file1 (DOCX 43 KB)

Supplementary file2 (DOCX 21 KB)

## Data Availability

Data have been extracted and synthesised from already published research studies.
