# Supplementary material for: Foodservice interventions and their influence on nutritional outcomes and satisfaction of adult oncology patients—a conceptual replication
Source: Support Care Cancer. 2025 Feb 24;33(3):217. doi: 10.1007/s00520-025-09264-5 (PMC11850487; doi:10.1007/s00520-025-09264-5)
Supplement: Supplementary file 1 — Supplementary file1 (DOCX 43 KB) [file 520_2025_9264_MOESM1_ESM.docx]

- 1. *neoplasms/ or exp *neoplasms by histologic type/ or exp *neoplasms by site/ or exp *neoplasms, hormone-dependent/ or exp *neoplasms, multiple primary/ or *neoplasms, post-traumatic/ or exp *neoplasms, radiation-induced/ or *neoplasms, second primary/ or Cancer Care Facilities/ or Oncology Service, Hospital/
  2. (neoplasm* or cancer* or oncolog* or tumor* or tumour* or glioma* or glioblastoma* or neoplastic* or leukaemia* or leukemia* or lymphoma* or malignant or malignancy or melanoma* or carcinoma* or chemotherapy or radiotherapy or radiation therapy).ti.
  3. 1 or 2
  4. nutrition therapy/ or exp diet therapy/ or nutritional support/ or enteral nutrition/ or food services/ or food service, hospital/ or menu planning/ or Nutritionists/ or Feeding Behavior/ or exp dietary supplements/ or food, fortified/ or Dietary Services/
  5. (food* or menu* or meal* or nutrition* or malnutrition or diet* or snack* or cook* or feed or feeding or eat* or cater* or red tray* or dining or supplements or calory or calories or kilojoule* or energy intake or enteral).ti.
  6. (food service* or food provision or food fortification or menu plan* or meal order* or meal distribution or fortified meal* or mealtime or meal time or nutritionist or nutritional support* or dietitian* or dietician* or diet therapist* or diet service* or hospital cater*).ti,ab.
  7. diet therapy.fs.
  8. 4 or 5 or 6 or 7
  9. Cancer Care Facilities/ or Oncology Service, Hospital/ or exp Hospitalization/ or inpatients/ or outpatients/ or exp Hospitals/ or exp Hospital Units/ or Food Service, Hospital/ or Ambulatory Care/ or ambulatory care facilities/ or exp outpatient clinics, hospital/ or Hospices/
  10. (hospital* or inpatient* or outpatient* or cancer cent* or cancer treatment cent* or cancer service* or ward* or oncology department* or oncology service* or ambulatory care or hospice*).ti,ab.
  11. 9 or 10
  12. 3 and 8 and 11
  13. (child* or pediatric* or paediatric* or infant*).ti.
  14. 12 not 13
  15. limit 14 to yr="2016 -Current"

*Supplementary figure 1:* Ovid Medline search strategy for systematic review of foodservice interventions in adult oncology patients 2016 onwards.
