# Supplementary material for: Foodservice interventions and their influence on nutritional outcomes and satisfaction of adult oncology patients—a conceptual replication
Source: Support Care Cancer. 2025 Feb 24;33(3):217. doi: 10.1007/s00520-025-09264-5 (PMC11850487; doi:10.1007/s00520-025-09264-5)
Supplement: Supplementary file 2 — Supplementary file2 (DOCX 21 KB) [file 520_2025_9264_MOESM2_ESM.docx]

**Ineligible intervention**

1. Kang WX, Li W, Huang SG, et al. Effects of nutritional intervention in head and neck cancer patients undergoing radiotherapy: a prospective randomized clinical trial. *Molecular and clinical oncology* 2016; 5: 279‐282. DOI: 10.3892/mco.2016.943.

2. González-Rodríguez M, Villar-Taibo R, Fernández-Pombo A, et al. Early versus conventional nutritional intervention in head and neck cancer patients before radiotherapy: benefits of a fast-track circuit. *European Journal of Clinical Nutrition* 2021; 75: 748-753. DOI: 10.1038/s41430-020-00786-1.

3. Shanthi S and Shambhavi. Effect of nutritional therapy on body mass index of cancer patients- A pilot study. *Journal of Clinical and Diagnostic Research* 2021; 15: LC16-LC19. DOI: 10.7860/JCDR/2021/49768.15555.

4. Hoang BV, Tran TT, Duong YT, et al. The Effects of Nutrition Intervention on Postoperative Patients with Tongue Cancer and Floor of Mouth Cancer. *Journal of Nutritional Science and Vitaminology* 2022; 68: 488-495. DOI: 10.3177/jnsv.68.488.

5. Britton B, Baker AL, Wolfenden L, et al. Eating As Treatment (EAT): A Stepped-Wedge, Randomized Controlled Trial of a Health Behavior Change Intervention Provided by Dietitians to Improve Nutrition in Patients With Head and Neck Cancer Undergoing Radiation Therapy (TROG 12.03). *International Journal of Radiation Oncology, Biology, Physics* 2019; 103: 353-362. DOI: 10.1016/j.ijrobp.2018.09.027.

6. Skouroliakou M, Grosomanidis D, Massara P, et al. Serum antioxidant capacity, biochemical profile and body composition of breast cancer survivors in a randomized Mediterranean dietary intervention study. *European journal of nutrition* 2018; 57: 2133‐2145. DOI: 10.1007/s00394-017-1489-9.

7. Ok JH, Lee H, Chung HY, et al. The potential use of a ketogenic diet in pancreatobiliary cancer patients after pancreatectomy. *Anticancer Research* 2018; 38: 6519-6527. DOI: 10.21873/anticanres.13017.

8. 马建红, 吴瑞臻, 叶正强, et al. 智能筛查系统与NRS-2002对头颈部肿瘤 住院病人营养风险筛查效果比较. *Chinese Nursing Research* 2021; 35: 3892-3896. DOI: 10.12102/j.issn.1009-6493.2021.21.025.

9. Nct. A Mixed Methods Study of Nutrition Practice in Cancer Care on Non-Hodgkin Lymphoma Population. *https://clinicaltrialsgov/show/NCT05376709* 2022.

10. Wang Y, Zhao D, Cao Y, et al. Nutritional counselling on weight loss and dietary intake of head and neck cancer patients undergoing radiotherapy: a historical control study for future intervention in china. *Clinical Nutrition ESPEN* 2021; 46: S722. DOI: 10.1016/j.clnesp.2021.09.505.

11. Lin JX, Chen XW, Chen ZH, et al. A multidisciplinary team approach for nutritional interventions conducted by specialist nurses in patients with advanced colorectal cancer undergoing chemotherapy: a clinical trial. *Medicine* 2017; 96: e7373. DOI: 10.1097/MD.0000000000007373.

12. Lu Q. Effect of whole-course nutrition management on patients with esophageal cancer undergoing concurrent chemoradiotherapy: a randomized control trial. *Annals of nutrition & metabolism* 2019; 75: 84‐. DOI: 10.1159/000501751.

13. Chen JH and Jin HW. Effect of evidence-based nursing combined with nutritional intervention on serum levels of il-6, il-8, and tnf-α in patients after combined laparoscopic-endoscopic radical surgery for rectal cancer. *World chinese journal of digestology* 2018; 26: 1137‐1143. DOI: 10.11569/wcjd.v26.i18.1137.

14. Artene DV and Blidaru A. Factors that influence oncology nutrition efficacy in breast cancer patients under antiestrogenic treatment. *Annals of oncology* 2018; 29: viii603‐viii604. DOI: 10.1093/annonc/mdy300.001.

15. Lu L, Chen X, Lu P, et al. Analysis of the Effect of Exercise Combined with Diet Intervention on Postoperative Quality of Life of Breast Cancer Patients. *Computational and mathematical methods in medicine* 2022; 2022. DOI: 10.1155/2022/4072832.

16. Orell H, Schwab U, Saarilahti K, et al. Nutritional counseling for head and neck cancer patients undergoing (chemo) radiotherapy-a prospective randomized trial. *Frontiers in nutrition* 2019; 6: 22. DOI: 10.3389/fnut.2019.00022.

17. Sittitrai P, Ruenmarkkaew D, Booyaprapa S, et al. Effect of a perioperative immune-enhancing diet in clean-contaminated head and neck cancer surgery: A randomized controlled trial. *International Journal of Surgery* 2021; 93: N.PAG-N.PAG. DOI: 10.1016/j.ijsu.2021.106051.

18. Aoyama T. Examining the Benefits of Digitally Selectable Meals Called "A La Carte Digital-Select" in Cancer Chemotherapy Patients. *Nutrition and metabolic insights* 2022; 15: 11786388221098507. DOI: https://dx.doi.org/10.1177/11786388221098507.

19. Najafi S, Haghighat S, Raji Lahiji M, et al. Randomized Study of the Effect of Dietary Counseling During Adjuvant Chemotherapy on Chemotherapy Induced Nausea and Vomiting, and Quality of Life in Patients With Breast Cancer. *Nutrition and cancer* 2019; 71: 575‐584. DOI: 10.1080/01635581.2018.1527375.

20. Ariza Cabrera E, Martín Estrada F, Polanco Muñoz M, et al. Benefts of the poor iodine diet in patients that are treated with 131-iodo for the treatment of thyroid neoplasms. *European Journal of Nuclear Medicine and Molecular Imaging* 2018; 45: S760-S761. DOI: 10.1007/s00259-018-4148-3.

21. Kämmerer U, Klement RJ, Joos FT, et al. Low Carb and Ketogenic Diets Increase Quality of Life, Physical Performance, Body Composition, and Metabolic Health of Women with Breast Cancer. *Nutrients* 2021; 13: 1029. DOI: 10.3390/nu13031029.

22. Kiss N, Gilliland S, Black J, et al. Improved efficiency and patient satisfaction following introduction of a nutrition assistant role in a head and neck cancer clinic. *Asia-Pacific Journal of Clinical Oncology* 2016; 12: 89.

23. Molassiotis A, Roberts S, Cheng HL, et al. Partnering with families to promote nutrition in cancer care: feasibility and acceptability of the PIcNIC intervention. *BMC Palliative Care* 2018; 17: 1-1. DOI: 10.1186/s12904-018-0306-4.

24. Tu YC, Ho XJ and Lin HY. The role of assessing nutritional status and applying adequate interventions in head and neck cancer patients treated with combined modalities. *Tzu Chi Medical Journal* 2017; 29: S20-S21.

25. de Souza APS, da Silva LC and Fayh APT. Nutritional Intervention Contributes to the Improvement of Symptoms Related to Quality of Life in Breast Cancer Patients Undergoing Neoadjuvant Chemotherapy: a Randomized Clinical Trial. *Nutrients* 2021; 13: 589‐589. DOI: 10.3390/nu13020589.

26. Tctr. Effect of perioperative immune-enhancing diet in head and neck cancer surgery patients: a randomized controlled trial. *https://trialsearchwhoint/Trial2aspx?TrialID=TCTR20200624003* 2020.

27. Zhang T, Huang H, Hu Q, et al. Clinical Efficacy of FOLFOX Chemotherapy Combined with Nutritional Support in the Treatment of Gastrointestinal Malignancies. *Anti-tumor pharmacy* 2018; 8: 635‐638 and 652. DOI: 10.3969/j.issn.2095-1264.2018.04.35.

28. Khodabakhshi A, Akbari ME, Mirzaei HR, et al. Feasibility, Safety, and Beneficial Effects of MCT-Based Ketogenic Diet for Breast Cancer Treatment: a Randomized Controlled Trial Study. *Nutrition and cancer* 2020; 72: 627‐634. DOI: 10.1080/01635581.2019.1650942.

29. Tung AC, Wen CF and Li PR. An evaluation on satisfaction with nutrition counseling for cancer inpatients. *FASEB Journal* 2016; 30.

30. Movahed S, Seilanian Toussi M, Pahlavani N, et al. Effects of medical nutrition therapy compared with general nutritional advice on nutritional status and nutrition-related complications in esophageal cancer patients receiving concurrent chemoradiation: a randomized controlled trial. *Mediterranean journal of nutrition and metabolism* 2020; 13: 265‐276. DOI: 10.3233/MNM-200424.

**Ineligible outcome**

1. Chen L, Zhao M, Tan L, et al. Effects of Five-Step Nutritional Interventions Conducted by a Multidisciplinary Care Team on Gastroenteric Cancer Patients Undergoing Chemotherapy: a Randomized Clinical Trial. Nutrition and cancer 2023; 75: 197‐206. DOI: 10.1080/01635581.2022.2104329.

2. Ijmker-Hemink V, Lize N, Beijer S, et al. Lessons learned from a randomized controlled trial on a home delivered meal service in advanced cancer patients undergoing chemotherapy: a pilot study. BMC nutrition 2021; 7. DOI: 10.1186/s40795-021-00407-5.

3. de Oliveira Faria S, Simões Lima GA, Lopes Carvalho A, et al. Clinically significant changes in health-related quality of life in head and neck cancer patients following intensive nutritional care during radiotherapy. European Journal of Oncology Nursing 2022; 56: N.PAG-N.PAG. DOI: 10.1016/j.ejon.2021.102065.

4. IJmker-Hemink V, Lize N, Beijer S, Raijmakers N, Wanten G, van den Berg M. Lessons learned from a randomized controlled trial on a home delivered meal service in advanced cancer patients undergoing chemotherapy: a pilot study. BMC nutrition. 2021 Dec;7(1):1-0.

**Ineligible population**

1. Musters SCW, van Noort HHJ, Bakker CA, et al. Impact of a surgical ward breakfast buffet on nutritional intake in postoperative patients: A prospective cohort pilot study. *PLoS ONE* 2022; 17. DOI: 10.1371/journal.pone.0267087.

2. Dijxhoorn DN, van den Berg MGA, Kievit W, et al. A novel in-hospital meal service improves protein and energy intake. *Clinical Nutrition* 2018; 37: 2238-2245. DOI: 10.1016/j.clnu.2017.10.025.

**Ineligible study design or output**

1. Deftereos I, Yeung JMC, Carter VM, et al. Nutritional Outcomes of patients Undergoing Resection for upper gastroIntestinal cancer in AuStralian Hospitals (NOURISH): protocol for a multicentre point prevalence study. BMJ open 2020; 10: e035824. DOI: https://dx.doi.org/10.1136/bmjopen-2019-035824.

2. Gany FM, Yorga S, Ramirez J, et al. Development of a Medically Tailored Hospital-based Food Pantry System. Journal of Health Care for the Poor & Underserved 2020; 31: 595-602. DOI: 10.1353/hpu.2020.0047.

3. Klement RJ and Sweeney RA. Impact of a ketogenic diet intervention during radiotherapy on body composition: I. Initial clinical experience with six prospectively studied patients. BMC research notes 2016; 9: 143. DOI: 10.1186/s13104-016-1959-9.

4. Nct. Effects of Nutritional Counseling on Nutritional Status and Quality of Life of Head and Neck Cancer Patients. https://clinicaltrialsgov/show/NCT03114202 2017.

5. Takata N, Kikuchi S, Kuroda S, et al. Effect of Patient-Participation Continuous Nutritional Counseling in Gastric Cancer Patients who Underwent Gastrectomy. Annals of surgical oncology 2023; 30: 1110-1118. DOI: https://dx.doi.org/10.1245/s10434-022-12572-3.

6. Kenny E, Touger‐Decker R and August DA. Structured Review of the Value Added by the Registered Dietitian to the Care of Gastrointestinal Cancer Patients. Nutrition in Clinical Practice 2021; 36: 606-628. DOI: 10.1002/ncp.10568.

7. Nct. The Effects of Whole Food Intervention on Mucositis in Patients Treated for Head and Neck Cancer. https://clinicaltrialsgov/show/NCT02575313 2015.

8. Nct. Eating Strategies for Chemotherapy Treatment. https://clinicaltrialsgov/show/NCT03010657 2017.

9. Ishaq O, Vega RM, Zullig L, et al. Food as medicine: a randomized controlled trial (RCT) of home delivered, medically tailored meals (HDMTM) on quality of life (QoL) in metastatic lung and noncolorectal GI cancer patients. Journal of clinical oncology 2019; 34: 155‐. DOI: 10.1200/jco.2016.34.26-suppl.155.

10. Kawabata H, Kitamura K, Yamamoto Y, et al. Availability of Frozen Foods and an In-Room Freezer for Terminally Ill Patients with Malignancies in a Palliative Care Unit. New Rochelle, New York: Mary Ann Liebert, Inc., 2020, p. 1419-1420.

11. Kct. Analysis of effects of nutritional interventions and nutritional needs in patients undergoing surgery of gastrointestinal cancer. https://trialsearchwhoint/Trial2aspx?TrialID=KCT0007250 2022.

12. Chi CI. The effect of whole-course educational and nutritional interventions on the nutritional status and compliance with chemotherapy of gastric cancer patients. https://trialsearchwhoint/Trial2aspx?TrialID=ChiCTR-IOQ-16010276 2016.

13. Purba M, Winarti H and Pangastuti R. Food intake and nutritional status of colorectal cancer patients undergoing radio-chemotherapy in Sardjito hospital. Annals of Oncology 2018; 29: v85-v86. DOI: 10.1093/annonc/mdy151.302.

14. Nct. High- Fiber/ Low-fat Diet for Prevention of Recurrent Clostridioides Difficile Infection in Oncology. https://clinicaltrialsgov/show/NCT04940468 2021.

15. Onions S and Wilderspin N. FOUR STAGE LOW FIBRE DIETARY GUIDANCE FOR PATIENTS SUFFERING SUBACUTE MALIGNANT BOWEL OBSTRUCTION. BMJ Supportive and Palliative Care 2021; 11: A58. DOI: 10.1136/spcare-2021-Hospice.153.

16. Musters S, van Noort H, van Dieren S, et al. Impact of a surgical ward breakfast buffet on nutritional intake in postoperative (oncological) patients. Annals of Oncology 2021; 32: S1260. DOI: 10.1016/j.annonc.2021.08.636.

17. Reuss-Borst M, Joos F, Klement R, et al. A low-carb diet improves metabolic parameters, BMI and body composition in breast cancer patients. Oncology Research and Treatment 2021; 44: 269. DOI: 10.1159/000518417.

18. Ford KL, Sawyer MB, Trottier CF, et al. Protein Recommendation to Increase Muscle (PRIMe): study protocol for a randomized controlled pilot trial investigating the feasibility of a high protein diet to halt loss of muscle mass in patients with colorectal cancer. Clinical Nutrition ESPEN 2021; 41: 175‐185. DOI: 10.1016/j.clnesp.2020.11.016.

19. Sánchez-Migallón Montull JM, Sendros Madroño MJ, Puig Piña R, et al. SUN-P109: Nutritional Intervention in Patients Undergoing Haematopetic Stem Cell Transplantation in the Catalan Institute of Oncology - Badalona. Clinical Nutrition 2017; 36: S94-S94. DOI: 10.1016/S0261-5614(17)30518-6.

20. Irct20171105037259N. Effect of ketogenic diet on patients with breast cancer. https://trialsearchwhoint/Trial2aspx?TrialID=IRCT20171105037259N2 2018.

21. Nct. Food as Medicine: an RCT to Study the Effect of Home Delivered, Medically Tailored Meals on Patients With Metastatic Cancer. https://clinicaltrialsgov/show/NCT02887235 2016.

22. Nct. Time Restricted Eating (TRE) Among Endometrial Cancer Patients. https://clinicaltrialsgov/show/NCT04783467 2021.

23. She G, Kuang X and Shi Y. Application of the Diet and Nutrition Management System in the Nutrition Management of Tumor Patients Treated with Chemotherapy. Anti-tumor pharmacy 2018; 8: 292‐295 and 300. DOI: 10.3969/j.issn.2095-1264.2018.02.38.

24. Shahid T, Kalyani N, Modak Das S, et al. To compare outcome of Intensive nutritional support with standard practise in head and neck cancer. Radiotherapy and oncology 2019; 133: S642‐. DOI: 10.1016/S0167-8140(19)31579-8.

25. Nct. The Impact of a Home Delivered Meal Service in Advanced Cancer Patients During Chemotherapy. https://clinicaltrialsgov/show/NCT03382171 2017.

26. Kct. The Safety and Feasibility of Postoperative Early Oral Nutrition after Total Gastrectomy for Gastric Carcinoma. https://trialsearchwhoint/Trial2aspx?TrialID=KCT0004707 2020.

27. Umin. Pilot study of the effect of nutritional intervention with elemental diet during chemotherapy for head and neck cancer. https://trialsearchwhoint/Trial2aspx?TrialID=JPRN-UMIN000012737 2013.

28. Nct. The Effect of Special Diets in Hematological Cancer Patients. https://clinicaltrialsgov/show/NCT02880709 2016.

29. lacone R, Scanzano C, De Caprio C, et al. SUN-P077: Nutritional Intervention in Hospitalized Cancer Patients. Clinical Nutrition 2016; 35: S72-S73. DOI: 10.1016/S0261-5614(16)30420-4.

30. Jensen SB, Larsen MK, Kjeldsen L, et al. Randomized controlled trial of liquid compared to solid food in patients with malignant hematologic disease. Clinical nutrition (Edinburgh, Scotland) 2016; 35: S181‐.

31. Nct. Application of Whole-course Standardized Nutrition Management During Peri-radiotherapy in Patients With Nasopharyngeal Carcinoma After Radiotherapy: a Multicenter Randomized Controlled Clinical Study. https://clinicaltrialsgov/show/NCT05008471 2021.

32. Nct. Nutritional Supplementation in Head and Neck Cancers. https://clinicaltrialsgov/show/NCT05379712 2022.

33. Nct. Effect of an Anti-inflammatory Diet on Patients With Cervical Cancer. https://clinicaltrialsgov/show/NCT03994055 2019.

34. Nct. Comparing Two Diets in Patients Undergoing HSCT or Remission Induction Chemo for Acute Leukemia and MDS (UF-BMT-LDND-101). https://clinicaltrialsgov/show/NCT03016130 2017.
